# Mitochondrial capacities and quality control following short‐ and long‐term weight restoration after simulated anorexia nervosa

**DOI:** 10.1113/EP093325

**Published:** 2025-11-29

**Authors:** Megan E. Rosa‐Caldwell, Toby L. Chambers, Lauren Breithaupt, Ruqaiza Muhyudin, Patience Salvalina Okoto, Sadie R. Thompson, Emily E. Rothacker, Claire Greenhill, Katie A. Wood, Kevin A. Murach, Sarah H. White‐Springer, Ursula B. Kaiser, Seward B. Rutkove

**Affiliations:** ^1^ Energy Availability and Muscle Metabolism Laboratory, Exercise Science Research Center, Department of Health Human Performance and Recreation University of Arkansas Fayetteville Arkansas USA; ^2^ Department of Neurology Beth Israel Deaconess Medical Center and Harvard Medical School Boston Massachusetts USA; ^3^ Molecular Muscle Mass Regulation Laboratory, Exercise Science Research Center, Department of Health Human Performance and Recreation University of Arkansas Fayetteville Arkansas USA; ^4^ Department of Psychiatry, Harvard Medical School, Eating Disorders Clinical and Research Program Massachusetts General Hospital Boston Massachusetts USA; ^5^ Mass General Brigham Multidisciplinary Eating Disorders Research Collaborative Mass General Brigham Boston Massachusetts USA; ^6^ Cachexia Research Laboratory, Exercise Science Research Center, Department of Health Human Performance and Recreation University of Arkansas Fayetteville Arkansas USA; ^7^ Arkansas Integrative Metabolic Research Center University of Arkansas Fayetteville Arkansas USA; ^8^ Equine Physiology, Department of Animal Science, College of Agriculture and Life Sciences; Department of Kinesiology & Sport Management, School of Education and Human Development Texas A&M University College Station Texas USA; ^9^ Division of Endocrinology, Diabetes and Hypertension Brigham and Women's Hospital and Harvard Medical School Boston Massachusetts USA

**Keywords:** mitochondrial biogenesis, mitochondrial dynamics, mitochondrial translation, mitophagy, muscle fatigability, starvation

## Abstract

Anorexia nervosa (AN) is a psychiatric disorder characterized by prolonged caloric restriction and skeletal muscle atrophy. Mitochondrial health is a key mediator of muscle function, yet the role of mitochondria during AN and following weight regain has not been investigated. The objective of this study was to evaluate mitochondrial capacities and quality control mechanisms in a rodent model of AN, spanning the acute underweight phase and multiple recovery periods. Through a series of experiments, 8‐week‐old female Sprague–Dawley rats underwent a 30‐day simulated AN protocol, followed by different durations of weight recovery via ad libitum feeding. Following designated interventions, muscle performance on a submaximal fatiguing protocol and components of mitochondrial function were evaluated. AN resulted in 23%–25% lower muscle performance compared to healthy controls, and these alterations remained even after short‐term weight gain. AN rats had 23% lower contribution of complex I to maximal mitochondrial electron transfer as well as alterations to genes important for mitochondrial translation and dynamics, many of which were not resolved with short‐term recovery. With long‐term recovery, muscle performance and mRNA content of genes related to mitochondrial translation were similar to healthy controls. However, genes related to mitochondrial fission were greater than healthy controls. AN results in reduced muscle performance during a fatiguing protocol, reliance on mitochondrial complex I and genes related to mitochondrial quality control. Many alterations persist with short‐term weight recovery; however, given sufficient time, many facets of mitochondrial health appear to normalize following AN, though there still may be long‐term consequences to mitochondrial dynamics.

## Abstract (highlights)

- **What is the central question of this study?**
  How does simulated anorexia nervosa (AN) affect skeletal muscle function and mitochondrial health during AN and following different durations of weight gain in a rodent model of AN?
- **What is the main finding and its importance?**
  Simulated AN in female rats leads to reduced muscle and mitochondrial respiratory capacities. Short‐term weight gain does not recover muscle function nor many aspects of mitochondrial health. Long‐term weight gain restores many aspects of muscle function and mitochondrial health, but some alterations to mitochondrial dynamics appear to remain.

## 1 INTRODUCTION

Anorexia nervosa (AN) is a severe psychiatric disorder marked by persistent food restriction and dangerously low body weight, affecting ∼1%–4% of women and ∼0.5% of men (Galmiche et al., 2019; Micali et al., 2017; Smink et al., 2013), with mortality risks ∼6 times greater than the general public (Arcelus et al., 2011). While often framed in terms of fat loss, individuals with AN also experience profound reductions in skeletal muscle mass and strength, pointing to a broader musculoskeletal phenotype that has received limited investigation. A recent systematic review of the clinical literature in humans suggests as much as ∼25% lower muscle mass and strength in those with AN compared to healthy controls (Rosa‐Caldwell et al., 2022). Moreover, AN often follows a chronic, relapsing course, with recovery trajectories extending up to 22 years from time of diagnosis (Eddy et al., 2017) and sustained recovery achieved by only ∼50% of individuals (Lock & Grange, 2019). Therefore, AN may represent one of the more persistent causes of muscle atrophy. Even when weight is restored, muscle mass appears to remain ∼9% lower than in healthy controls (Rosa‐Caldwell et al., 2022). One study found significant differences in muscle size and force production in patients 27 years post‐recovery from AN relative to controls (Mueller et al., 2015). Despite these musculoskeletal complications and the prevalence of the disease, little is known about skeletal muscle physiology across the course of AN, including the acute underweight state, early refeeding and longer‐term recovery.

Mitochondrial function is a significant mediator of cellular health, particularly in energetically demanding tissues like skeletal muscle. Mitochondrial function, including oxygen uptake and ATP production, generation of reactive oxygen species, and mitochondrial quality control mechanisms are known to be altered across a variety of muscle pathologies (Brown et al., 2017; Greene et al., 2015; Lee et al., 2019; Rosa‐Caldwell et al., 2021). Mitochondrial quality control refers to processes that facilitate overall mitochondrial efficiency and function. Broadly, the various mitochondrial quality control mechanisms include: biogenesis (synthesis of new mitochondria), dynamics (fusion of mitochondria to share components of the electron transfer chain to improve overall mitochondrial efficiency, and fission of unhealthy mitochondrial components away from the mitochondrial network), mitochondrial translation (translation of mitochondria‐specific genes encoded within mitochondrial DNA), and mitophagy (autophagic destruction of mitochondria, typically unhealthy/dysfunctional mitochondria) (Yan et al., 2012). Beneficial interventions such as exercise can shift various mitochondrial control mechanisms such as fusion to elicit greater mitochondrial efficiency (Lee et al., 2014; Li et al., 2025; Ruegsegger et al., 2023), whereas pathological conditions such as insulin resistance can alter mitochondrial quality control mechanisms towards greater mitochondrial fragmentation (dynamics) and accumulation of dysfunctional mitochondria (mitophagy) (Jheng et al., 2012; Rosen et al., 2025). Given females rely more on oxidative metabolism (and hence mitochondrial activity) compared to males (Rosa‐Caldwell & Greene, 2019) and AN disproportionally affects females (Galmiche et al., 2019; Micali et al., 2017; Smink et al., 2013), mitochondrial dysfunction may play a particularly important role in persistent musculoskeletal deficits during AN and attempted weight restoration.

Prior research in human clinical subjects and in rodent models suggests that AN is associated with mitochondrial dysfunction systemically and across multiple tissues. In the central nervous system, studies in humans and rodents have noted lower mitochondrial content markers (Gaiaschi et al., 2024), lower mitochondrial respiration (Lindfors et al., 2011), and a shift in mitochondrial dynamics towards greater fission (Bhasin et al., 2023), resulting in increased mitochondrial fragmentation and correspondingly a less efficient mitochondrial network. Of note, a greater shift towards mitochondrial fission was reported in the amygdala of AN mice following weight restoration, potentially suggesting that mitochondrial alterations may play a role in AN relapse and resistance to treatment (Bhasin et al., 2023). Systemically, circulating cells from patients with AN, such as leukocytes and peripheral blood mononuclear cells (PBMCs), have lower mitochondrial respiration, driven by complex I activity, lower mitochondrial density, and greater reactive oxygen species production (Omodei et al., 2015; Victor et al., 2014). In aggregate, these results suggest that AN is associated with multiple alterations to mitochondrial function. The effects of AN on skeletal muscle mitochondria and how these changes relate to clinically meaningful recovery trajectories remain poorly understood. The purpose of this study was to evaluate multiple components of mitochondrial function and quality control mechanisms in a rodent model of AN, spanning the acute underweight phase and recovery periods approximating early (∼5 months), intermediate (∼15 months), and long‐term (∼2.5 years) human recovery. We hypothesized that simulated AN would result in lower mitochondrial efficiency and alterations to mitochondrial quality control processes, and that these disruptions would persist across recovery phases, even after weight restoration.

## 2 METHODS

### 2.1 Ethical approval

All animal experiments were approved by either University of Arkansas or Beth Israel Deaconess Medical Centre Institutional Animal Care and Use Committees [AUP: 24011 (experiment 1) and 009‐2022 (experiments 2 and 3), respectively] and conducted in accordance with respective institutions’ animal welfare guidelines. All animal experiments were conducted in accordance with *Experimental Physiology*’s policies regarding animal experiments and adhere to ARRIVE guidelines 2.0.

### 2.2 Animal experiments

**FIGURE 1:**
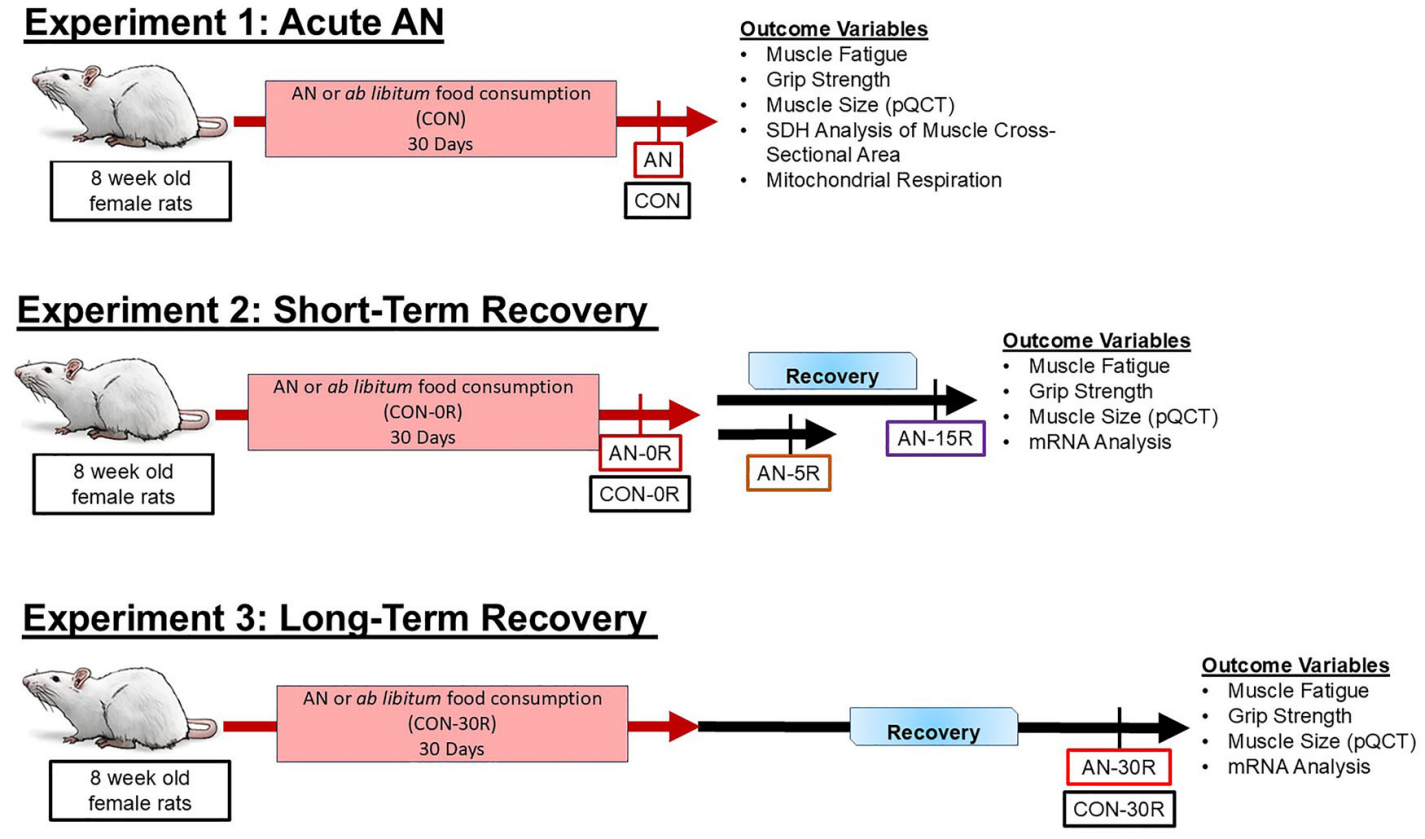
Experimental design for three separate animal experiments. Experiment 1: *n* = 9/group (18 rats total). AN, simulated anorexia nervosa; CON, healthy controls. Experiment 2: *n* = 11/group (44 rats total). CON‐0R, healthy controls; AN‐0R, simulated anorexia nervosa without any recovery; AN‐5R, simulated anorexia nervosa followed by 5 days of weight recovery, AN‐15R, simulated anorexia nervosa followed by 15 days of weight recovery. Experiment 3: *n* = 11/group (22 rats total). CON‐30R, healthy controls age‐matched to AN‐30R; AN‐30R, simulated anorexia nervosa followed by 30 days of weight recovery.

All animal experiments were conducted in temperature (∼23°C) and humidity‐controlled animal facilities with 12:12 h light–dark cycles. Following designated interventions for all experiments, rats were anaesthetized with 3%–4% isoflurane, and skeletal muscle was collected and processed for further analysis (either prepared for mitochondrial respiration assays as described below or snap‐frozen in liquid nitrogen). The gastrocnemius muscle was chosen for biochemical and histological analysis because our muscle function data (fatiguing protocol and in vivo muscle size) are largely driven by the gastrocnemius. After muscle collection, rats were euthanized by cardiac puncture. For all experiments, feeding status was matched across rats (food provided ∼90 min prior to euthanasia). Euthanasia procedures were aligned with the 2020 American Veterinary Medical Association Guidelines on Euthanasia. Depictions of individual animal experiments are shown in Figure 1.

### 2.3 Anorexia nervosa procedure

Simulated AN was completed for experiments 1–3 using our previously described model (Rosa‐Caldwell et al., 2025). Eight‐week‐old female Sprague–Dawley rats were provided with 40%–50% less food than their baseline ad libitum consumption. Eight weeks of age roughly corresponds to late adolescence in rats, which is approximately the modal age of onset for AN (Hudson et al., 2007; Stice et al., 2013). Rats were moved briefly from group housing without food to single housing for a 2‐h period daily during which time food was provided (∼6–8 g, ∼40%–50% less than ad libitum consumption measured prior to AN protocol). After either 2 h or complete consumption of food, rats were moved back to group housing. Rats continued this feeding intervention for 30 continuous days. Rats had ad libitum access to water throughout AN (or control) interventions. With this model, we recapitulate many of the clinical features of AN such as altered oestrous cycles, lower bone mineral density and lower body fat (Rosa‐Caldwell et al., 2025), while allowing for the development of sustained lower body weight over 30 days, corresponding to roughly 2–3 years of illness in humans (Minnes et al., 2024; Wang et al., 2025; Wu et al., 2024).

#### 2.3.1 Experiment 1: acute AN

Simulated AN was induced in female Sprague–Dawley rats purchased from Inotiv (Indianapolis, IN, USA, *n* = 9) for 30 days. Ad libitum fed age‐matched rats served as healthy controls (*n* = 9).

#### 2.3.2 Experiment 2: acute AN and short‐term recovery

Simulated AN was induced as described above (rats were purchased from Charles River, Wilmington, MA, USA). Then, rats were divided into acute AN with: (1) no recovery (AN‐0R, *n* = 11); (2) 5 days of recovery (AN‐5R, *n* = 11); or (3) 15 days of recovery (AN‐15R, *n* = 11). These timelines correspond roughly to ∼5 months and ∼15 months of recovery, respectively (Minnes et al., 2024; Wang et al., 2025; Wu et al., 2024). For recovery interventions, rats were provided food ad libitum for the designated recovery periods. Ad libitum‐fed rats, age‐matched to AN‐0R rats, served as healthy controls (*CO‐N0R, n* = 11).

#### 2.3.3 Experiment 3: acute AN and long‐term recovery

Simulated AN was again induced as described, after which rats were provided with food ad libitum for 30 days to allow weight recovery, matching the duration of recovery to the duration of simulated AN, to model chronic effects of previous AN (AN‐30R, *n* = 11). Age‐matched ad libitum‐fed rats served as healthy controls (CO‐N30R, *n* = 11). Rats were purchased from Charles River (Wilmington, MA).

### 2.4 Muscle functional and size assessments

#### 2.4.1 Grip strength

Rear paw grip strength was assessed as previously described (Rosa‐Caldwell et al., 2023a, 2023b). Briefly, rats were held gently and their rear paws placed on a grip bar attached to a force transducer (Harvard Biosciences, Inc., Holliston MA). Rats were then pulled away from the bar until they released it, and the corresponding force was recorded. Rats completed three trials, and the average force generated was used for analysis. Grip strength was assessed before interventions and prior to euthanasia.

#### 2.4.2 Muscle area via peripheral quantitative computed tomography

Muscle area of the lower leg (primarily gastrocnemius muscle) was assessed as we have described previously (Rosa‐Caldwell et al., 2023a, 2023b). Briefly, rats were anaesthetized with 2%–3% isoflurane and placed on a heating pad (Kent Scientific, Torrington, CT, RightTemp Jr warming system). Their left legs were then extended and gently taped to a rat‐specific holder for peripheral quantitative computed tomography (pQCT). pQCT measurements (Stratee, XCT Research SA+, Pforzheim, Germany) were collected 2.00 mm from the tibial plateau, distal to the growth plate. pQCT measurements were collected with the following parameters: voxel size: 0.10 mm and CT speed of: 10 mm/s.

#### 2.4.3 Plantar flexion muscle performance to a fatiguing stimuli

Rats were anaesthetized with 2%–3% isoflurane and placed on a heating pad. The left leg was then gently taped to a foot plate attached to a force transducer (Aurora Scientific, Ontario, Canada). Needle electrodes were placed in the popliteal fossa to elicit plantar flexion, and location was tested with a small (10 Hz) twitch stimulation. Once appropriate needle location was confirmed, the plantar flexor muscles were stimulated with a series of contractions to elicit muscle fatigue. Contractions were 200 ms stimulations at 40 Hz, with 800 ms of rest between stimulations (1 contraction/s), for a total of 2 min, resulting in 120 contractions. Muscle performance curves were generated for each animal; from each curve, area under the curve (AUC), time to 50% force production and peak force achieved were quantified.

### 2.5 Mitochondrial respiratory capacities

Mitochondrial oxidative phosphorylation (P) and electron transfer (e) capacities were quantified via high‐resolution respirometry as described previously (Artman et al., 2024; Latham et al., 2019; Wesolowski et al., 2023). Briefly, a small portion of the left gastrocnemius muscle was removed and placed in ice‐cold BIOPS buffer (10 mM Ca‐EGTA buffer, 0.1 µM free calcium, 20 mM imidazole, 20 mM taurine, 50 mM K‐MES, 0.5 mM dithiothreitol, 6.56 mM MgCl_2_, 5.77 mM ATP and 15 mM phosphocreatine; pH 7.1). Care was taken to ensure consistency across all samples on the approximate location of sample acquisition from the gastrocnemius (i.e. lateral/proximal portion). Muscle fibres were then gently teased apart while in the BIOPS buffer on ice using fine tip forceps. Once samples were teased apart, they were incubated on a rotary tube mixer (RotoFlex R2000; Cole‐Palmer; Vernon Hills, IL, USA) in 1 mL fresh BIOPS containing 50 µg/mL saponin for 30 min at 4°C (Millipore Sigma, Burlingon, MA, USA; cat. no. 84510). Samples were then rinsed in mitochondrial respiration solution (Mir05: 110 mM sucrose, 60 mM potassium lactobionate, 0.5 mM EGTA, 3 mM MgCl2∙6H_2_O, 20 mM taurine, 10 mM KH_2_PO_4_, 20 mM HEPES, 1 g/L BSA, pH 7.1) for an additional 10 min at 4°C. Following this incubation, samples were weighed (∼2.00–4.0 mg) and placed in a chamber of an Oroboros Oxygraph‐2k (O2k; Oroboros, Innsbruck, Austria), containing MiR06 (MiR05 + 280 U/mL catalase) and with 20 mM creatine. Samples then underwent a substrate–uncoupler–inhibitor titration protocol, as performed previously (Lark et al., 2016). The procedure includes: (1) complex I (CI) substrates pyruvate (5 mM) and malate (1 mM), to determine non‐phosphorylating proton leak (leak); (2) ADP (2.5 mM), to quantify complex I‐supported P (P_CI_); (3) glutamate (10 mM), an additional complex I substrate; (4) cytochrome *c* (10 µM), to measure integrity of the outer mitochondrial membrane; (5) the complex II substrate succinate (10 mM), to measure maximal coupled P (P_CI+II_); (6) uncoupler carbonyl cyanide 3‐chlorophenylhydrazone (CCCP, 0.5 µM steps), to attain maximal non‐coupled E (E_CI+II_); (7) a complex I inhibitor, rotenone (0.5 µM), to measure complex II‐supported electron transfer (E_CII_); and (8) a complex III inhibitor, antimycin A (2.5 µM), to quantify non‐mitochondrial residual O_2_ consumption (ROX). All data were corrected for ROX. Throughout the experiment, chambers were kept at 38°C and in hyperoxic conditions (200–650 µM O_2_). If the O_2_ concentration in the chamber started dropping below 200 µM, 200 mM H_2_O_2_ was added to the chamber to increase the O_2_ concentration. Data were analysed relative to tissue weight (integrative) or as a ratio to maximal electron transfer capacity E_CI+II_ (flux control ratio, FCR). Sample FCR was calculated by dividing the flux in each respiratory state by the sample's maximal flux during non‐coupled respiration (E_CI+II_), thus calculating the relative contribution of each complex (complex I or II) to maximal electron transfer capacity (E_CI+II_). Due to experimental limitations, only a subset of samples were analysed and presented (*n* = 4–6/group).

### 2.6 Succinate dehydrogenase staining and analysis

Succinate dehydrogenase (SDH) staining was completed as previously described (Rosa‐Caldwell et al., 2017). Following snap‐freezing of whole gastrocnemius muscles in liquid nitrogen, cross‐sectional slices at 10 µm thickness were collected at the mid‐belly of the gastrocnemius with an Epredia HM 525NX cryostat (Kalamazoo, MI). Once cut, sections were placed on charged microscope slides and kept frozen at −80°C until staining. For staining, sections were incubated in SDH staining solution containing [50 mM sodium succinate, 50 mM phosphate buffer (0.12 M KH_2_PO_4_ and 0.88 M Na_2_HPO_4_), 0.5 mg/mL Nitroblue tetrazolium] at 37°C for 40 min. Slides were then rinsed in dH_2_O for 3 min and mounted with glycerol‐based mounting medium and a coverslip. Images were collected with a white light microscope and associated microscope software (CX43 Upright Microscope and cellSens Standard Version 4.2, Hunt Optics & Imaging, Pittsburgh, PA). The entire cross‐section of the gastrocnemius was imaged; only portions of the sample without freeze damage were quantified. Image analysis was conducted with ImageJ software. First, a blinded investigator categorized muscle fibres as SDH positive (SDH^+^, purple) or SDH negative (SDH^−^, not purple). Then the cross‐sectional area (CSA) of SDH^+^ and SDH^−^ fibres was measured by the same blinded investigator. Average CSA of SDH^+^ and SDH^−^ fibres were quantified for each rat (∼400 fibres per rat) and used for final analysis. Due to experimental limitations, a subset of samples were analysed and presented (*n* = 5–6/group).

### 2.7 mRNA analysis by real time quantitative PCR (RT‐qPCR)

RNA was isolated from homogenized gastrocnemius samples as previously described (Greene et al., 2015), using a commercial kit (Purelink RNA Mini Kit, Thermo Fisher Scientific, cat. no. 12183025). Following RNA isolation, samples were assessed for RNA concentration and quality (sufficient quality defined as 260/280 ratio > 2.0). RNA was then reverse transcribed to cDNA using a commercial kit (Superscript IV VILO, Thermo Fisher Scientific, Waltham, MA, USA, cat. no. 11756500). cDNA was diluted to 0.5 ng/mL and analysed using TaqMan Master Mix (TaqMan Fast Advance Master Mix, Thermo Fisher Scientific, cat. no. 4444965) or SYBR Master Mix (Thermo Fisher Scientific, cat. no. 4364346) as previously described, using the $2^{-\Delta \Delta C_{t}}$ method (Greene et al., 2015). The lists of TaqMan probes and primer sequences are found in Tables 1 and 2, respectively. Primers used in this study have been validated previously (Rosa‐Caldwell et al., 2019). All data were normalized to *Hprt*, a housekeeping gene, which did not differ between any groups analysed in the study (*P *> 0.05).

**TABLE 1:** TaqMan probes.

Gene	Probe number
*Pgc1α1*	Rn01749553_g1
*Opa1*	Rn00592200_m1
*Drip1*	Rn00586466_m1
*Tfam*	Rn00580051_m1
*Mfn1*	Rn00594496_m1
*Hprt*	Rn01527840_m1

**TABLE 2:** SYBR primers.

**Gene**	**Forward primer**	**Reverse primer**	**Ref Seq ID no**.
*Mfn2*	*AGGCGATTTGAGGAGTGCAT*	*ACAATAAACCCGCTGCTCCT*	NM_130894.4
*Mff*	*AAGCGAAGAGATCCGAGCAG*	*CCTCTGACGCTCCTTCAACA*	NM_001039015.2
*Bnip3*	*AAGCGCACAGCTACTCTCAG*	*ACGCCTTCCAATGTAGATCCC*	NM_053420.3
*mtIF2*	*GCCAAGGTCCCCAGTTGTTA*	*CTGCCACTTGAGTTTCCCGA*	NM_001004254.1
*mtIF3*	*GGACAGCATGATTTGGACACC*	*TCTCATCCATCTCACTCCCG*	NM_001115041.2
*Tufm*	*GCCTCCAGGGAAGGAACTTG*	*GTGCCAATGGTCTTGTTGCC*	NM_001106295.1
*Taco1*	*CTTAGAGGTGTGTCGCAGCA*	*CCACGACCCTCATACAGCAA*	NM_001108302.1

### 2.8 Statistical analysis

Prior to analysis all data were assessed for normality and skew. Data points that were >2 standard deviations from the mean were removed as outliers. For experiments that involved two groups (experiments 1 and 3), data were analysed by Student's *t*‐test, with significance defined as *P <* 0.05. For variables in which baseline differences were thought to potentially contribute to final post‐intervention testing (e.g. muscle function data, tissue masses, or muscle fibre cross‐sectional area), a covariate of either baseline values (for repeated measurements) or baseline bodyweights were applied. For experiments with more than one group (i.e. experiment 2), data were analysed by one‐way ANOVA with Dunnett's *post hoc* adjustment to compare experimental groups (AN‐0R, AN‐5R, or AN‐15R) to healthy controls (CON‐0R). Significance was denoted as a Dunnett‐adjusted *P <* 0.05. Similar to experiments 1 and 3, for variables where baseline differences were thought to contribute to final post‐intervention testing, a covariate of either baseline values or baseline bodyweights was applied. All data were analysed by SAS (SAS Studio Release: 3.81, SAS Institute Inc., Cary, NC, USA). All data, associated statistical code and data outputs are deposited on the Open Science Framework page for this project at https://doi.org/10.17605/OSF.IO/GJ5EF.

## 3 RESULTS

### 3.1 Experiment 1: simulated anorexia nervosa (AN) resulted in reductions to skeletal muscle function

**FIGURE 2:**
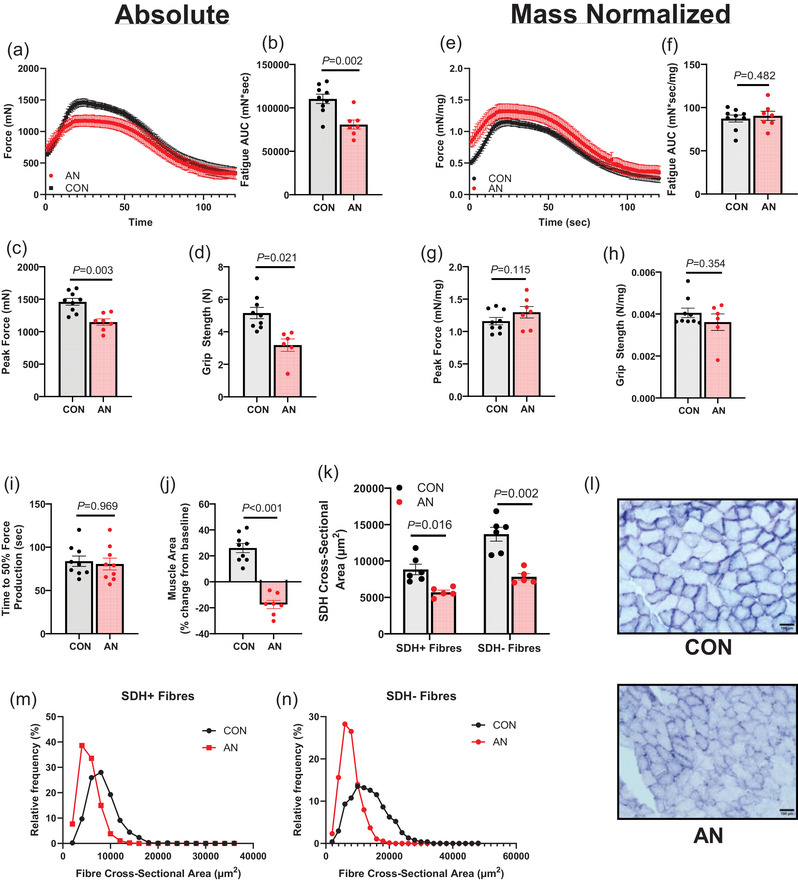
Muscle function results from experiment 1 (Acute AN). (a) Absolute force fatigue curve with 1 contraction/s for 120 s. (b) Absolute area under the curve (AUC) from fatigue curve. (c) Absolute peak force achieved during fatigability protocol. (d) Mean absolute grip strength. (e) Gastrocnemius mass normalized force fatigue curve with 1 contraction/s for 120 s. (f) Gastrocnemius mass normalized area under the curve (AUC) from fatigue curve. (g) Gastrocnemius mass normalized peak force achieved during fatigability protocol. (h) Gastrocnemius mass normalized mean grip strength. (i) Time to 50% of maximal force production during fatigue curve. (j) Muscle area (percentage change from baseline) of the lower leg assessed by pQCT. (k) Average muscle fibre cross‐sectional area (CSA) of succinate dehydrogenase (SDH) positive and negative fibres. (l) Representative SDH images from AN and CON rats. (m) Histogram of muscle fibre CSA for SDH positive fibres. (n) Histogram of muscle fibre CSA for SDH negative fibres. Quantitative data are depicted as means ± SEM. Data were analysed by *t*‐test with a covariate of either baseline values (fatigue data) or baseline bodyweight (muscle CSA data).

Similar to our previous reports (Rosa‐Caldwell et al., 2025), we observed marked changes in bodyweight and tissue mass of several hindlimb skeletal muscles (Table 3). Additionally, unadjusted muscle force production during a fatiguing protocol of the plantar flexor muscles was lower in AN compared to CON (Figure 2a). This change in submaximal muscle function was reflected in 25% lower area under the curve (AUC) of the muscle fatigability curve (*P =* 0.002, Figure 2b) and 22% lower peak force achieved during the muscle fatigability assessment (*P =* 0.003, Figure 2c). Raw average grip was 40% lower in AN compared to CON (*P =* 0.021, Figure 2e). To evaluate muscle quality, defined as muscle strength relative to amount of muscle, we normalized our fatigue and grip strength parameters to gastrocnemius mass. With this normalization, there were no differences between AN and CON on fatigue curves (Figure 2e), fatigue AUC (*P =* 0.482, Figure 2f), peak force (*P =* 0.118, Figure 2g) or grip strength (*P =* 0.384, Figure 2h). Overall time to 50% force production (which would be the same for raw or normalized data) was not different between AN and CON (*P =* 0.969, Figure 2i). With regards to muscle size, CON rats had ∼20% muscle growth over the course of the 30‐day intervention, while AN rats had ∼18% muscle loss over the course of the intervention (*P <* 0.001, Figure 2j). Moreover, muscle cross‐sectional area (CSA) of SDH positive fibres was 35% lower in AN compared to CON, resulting in a shift of muscle fibre distribution towards smaller fibres (*P =* 0.016, Figure 2k–n). CSA of SDH negative fibres was 43% lower in AN compared to CON, with a corresponding shift towards smaller muscle fibres (*P =* 0.002, Figure 2k–n).

**TABLE 3:** Muscle weights following acute AN (experiment 1).

	CON	AN
Bodyweight, baseline (g)	177.9 ± 5.4	193.5 ± 5.4
Bodyweight, post (g)	227.87 ± 2.9	145.9 ± 2.9*
Gastrocnemius (g)	1.30 ± 0.30	0.86 ± 0.30*
Plantaris (mg)	252.41 ± 5.65	174.12 ± 6.03*
Soleus (mg)	105.64 ± 3.67	81.57 ± 3.67*
Tibialis anterior (mg)	470.90 ± 12.87	319.38 ± 13.74*
Extensor digitorum longus (mg)	93.28 ± 4.26	69.88 ± 4.26*

Data are depicted as means ± SEM, *n* = 8–9/group, *P < 0.05 vs. CON. Data are corrected with covariate of baseline bodyweight. Abbreviations: CON, healthy control age‐matched to AN; AN, simulated anorexia nervosa.

### 3.2 Experiment 1: AN did not alter tissue‐weight normalized respiratory capacities, but did affect some aspects of mitochondrial flux control ratios compared to CON

**FIGURE 3:**
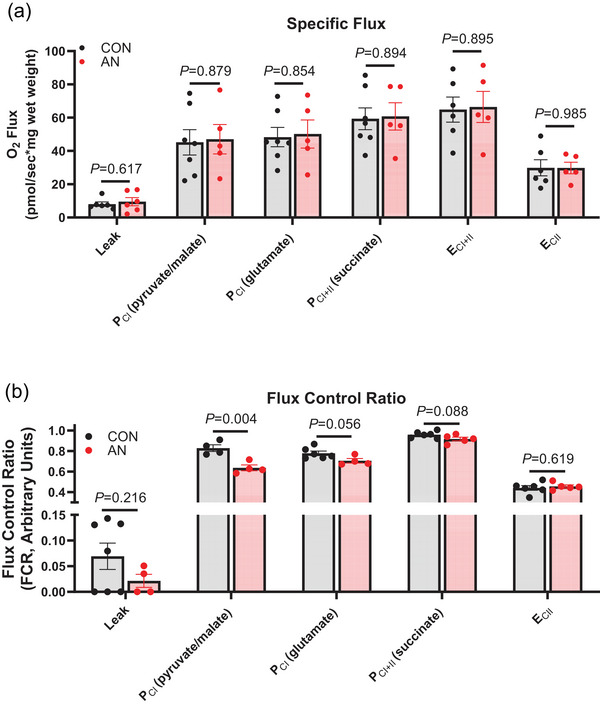
Mitochondrial respiration data from experiment 1 (Acute AN). (a) Oxygen (O_2_) specific flux relative to tissue weight. (b) O_2_ flux relative to electron transfer through complex I + II (flux control ratio; FCR). Data are depicted as means ± SEM. Data analysed by *t*‐test.

There were no differences between CON and AN on leak respiration (*P =* 0.617), phosphorylating respiration through Complex I (P_CI_) stimulated by pyruvate/malate (*P =* 0.879) and with the addition of glutamate (*P =* 0.854), maximal phosphorylating respiration through complexes I and II (P_CI+II_; *P =* 0.894), maximal non‐coupled electron transfer (E_CI+II_; *P =* 0.895), or complex II‐supported electron transfer (E_CII_; *P =* 0.985, Figure 3a). The flux control ratio (FCR), which provides insight into the contribution of each complex to maximal electron transfer capacity (E_CI+II_), did not differ between AN and CON for leak respiration (*P =* 0.216); however, FCR_PCI_ stimulated with pyruvate/malate in AN rats was ∼23% lower (*P =* 0.004, Figure 3b) and ∼10% lower with the addition of glutamate (*P =* 0.056, Figure 3b). There were no differences in FCR when complex I + II were combined (FCR_PCI+II_), though AN rats did have ∼5% lower FCR compared to CON (*P =* 0.088, Figure 2b), suggesting lower coupling of oxidative phosphorylation and electron transfer in AN. There were no differences between groups for FCR_ECII_ (*P =* 0.619, Figure 3b).

### 3.3 Experiments 2 and 3: AN induced changes to skeletal muscle performance, which did not recover during short‐term recovery; however, many facets of muscle performance did resolve over long‐term recovery

**FIGURE 4:**
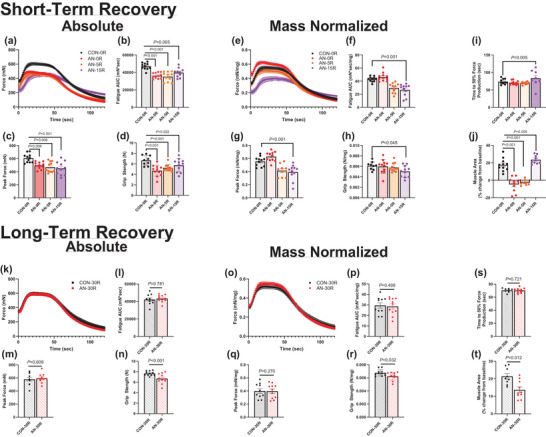
Muscle function data from experiment 2 (short‐term recovery) and experiment 3 (long‐term recovery). (a) Absolute values fatigue curve with 1 contraction/s for 120 s from experiment 2. (b) Absolute force area under the curve (AUC) derived from the fatigue curve from experiment 2 (shown in Figure 4a). (c) Absolute peak force achieved during fatigue protocol from experiment 2. (d) Absolute mean rear paw grip strength from experiment 2. (e) Gastrocnemius mass normalized fatigue curve with 1 contraction/s for 120 s from experiment 2. (f) Gastrocnemius mass normalized area under the curve (AUC) derived from the fatigue curve from experiment 2. (g) Gastrocnemius mass normalized peak force achieved during fatigue protocol from experiment 2. (h) Gastrocnemius mass normalized mean rear paw grip strength from experiment 2. (i) Time to 50% of maximal force production during fatigue curve from experiment 2. (j) Muscle area (percentage change from baseline) of the lower leg assessed by pQCT in experiment 2. (k) Absolute force fatigue curve with 1 contraction/s for 120 s from experiment 3. (l) Absolute force area under the curve (AUC) from fatigue curve from experiment 3. (m) Absolute peak force achieved during fatiguing protocol from experiment 3. (n) Absolute mean grip strength from experiment 3. (o) Gastrocnemius mass normalized fatigue curve with 1 contraction/s for 120 s from experiment 3. (p) Gastrocnemius mass normalized area under the curve (AUC) derived from the fatigue curve from experiment 3. (q) Gastrocnemius mass normalized peak force achieved during fatiguing protocol from experiment 3. (r) Gastrocnemius mass normalized average grip strength from experiment 3. (s) Time to 50% of maximal force production during fatigue curve from experiment 3. (t) Muscle area (percent change from baseline) of the lower leg assessed by pQCT in experiment 3). Data are depicted as means ± SEM. Data were analysed by one‐way ANOVA with Dunnett's *post hoc* adjustment (experiment 2) or *t*‐test (experiment 3). Baseline values were used as covariates.

Phenotypic muscle data for these rats have been published previously (Rosa‐Caldwell et al., 2025). Briefly, we noted lower muscle masses with AN‐0R (∼26% lower gastrocnemius, ∼45% lower soleus, ∼22% lower plantaris, ∼22% lower tibialis anterior compared to CON‐0R), which gradually increased in AN‐5R (∼22% lower gastrocnemius, ∼15% lower soleus, ∼20% lower plantaris, ∼13% lower tibialis anterior compared to CON‐0R) and AN‐15R (no difference in gastrocnemius, ∼8% lower soleus, ∼3% larger plantaris, ∼9% greater tibialis anterior compared to CON‐0R). In rats after long‐term recovery, we found that the mass of gastrocnemius muscles was 9% lower in AN‐30R compared to CON‐30R (*P <* 0.05). Similar to experiment 1, AN‐0R resulted in reduced raw muscle function during a submaximal fatiguing protocol. In our short‐term recovery cohorts (AN‐5R and AN‐15R), we found that absolute muscle function remained lower compared to CON‐0R (Figure 4a). In particular, the AUC for the endurance curves were lower by 23%, 23%, and 15% in AN‐0R, AN‐5R and AN‐15R, respectively, compared to CON‐0R (*P <* 0.001, *P <* 0.001 and *P =* 0.005, respectively, Figure 4b). Peak forces achieved during the fatigue protocol were also lower, with AN‐0R rats having 18%, AN‐5R rats 19% and AN‐15R rats 26% lower peak force, compared to CON‐0R (*P =* 0.008, *P =* 0.008 and *P <* 0.001, respectively, Figure 4c). Additionally, AN‐0R, AN‐5R and AN‐15R had ∼24%, ∼19% and ∼16% lower average grip strength compared to CON‐0R, respectively (*P <* 0.001, *P <* 0.001 and *P =* 0.002, respectively, Figure 4d).

When muscle function data were normalized to gastrocnemius mass, differences in functional parameters between AN‐0R and CON‐0R were generally lost. AN‐15R had ∼38% lower mass normalized fatigue AUC (*P =* 0.001, Figure 4f). Similarly, AN‐15R had ∼30% lower peak force achieved during a fatiguing protocol when normalized to gastrocnemius mass compared to CON‐0R (*P <* 0.001, Figure 4g). AN‐15R rats also had ∼18% lower average grip strength normalized to gastrocnemius mass (*P =* 0.048, Figure 4h). AN‐15R had had slightly longer time to 50% force production compared to CON‐0R (16%, *P =* 0.005, Figure 4i), with no other differences noted between CON‐0R and AN‐0R or AN‐5R (*P =* 0.806 and *P =* 0.438, respectively).With regards to muscle size, AN‐0R and AN‐5R rats had lower muscle area change compared to CON‐0R (∼10% lower for both compared to ∼20% growth in CON‐0R, *P <* 0.001, Figure 4j). However, by 15 days of recovery (AN‐15R), muscle area had exceeded CON‐0R (*P =* 0.005, Figure 4j).

When recovery was extended to 30 days (matching the amount of time in simulated AN with the time in recovery), there were no differences in muscle function to a fatiguing protocol between AN‐30R and CON‐30R, either absolute or mass normalized values (Figure 4k and m). This was quantified with no difference in the fatigue curve AUC (*P =* 0.781, Figure 4l (absolute) and *P =* 0.498, Figure 4n (mass normalized)), peak force during the fatiguing protocol (*P =* 0.609, Figure 4o, *P* = 0.270, Figure 4q), or time to 50% force production (*P =* 0.721, Figure 4s). However, absolute average grip strength was ∼15% lower in AN‐30R compared to CON‐30R (*P <* 0.001, Figure 4p) and normalized average grip strength was ∼8% lower in AN‐30R compared to CON‐30R (*P =* 0.032, Figure 4r). Finally, muscle area change from baseline remained lower in AN‐30R compared to CON‐30R, with CON‐30R rats having ∼20% growth during the interventions and AN‐30R rats having ∼15% growth during the interventions (*P =* 0.012, Figure 4t).

### 3.4 Experiments 2 and 3: Simulated AN induced changes to genes moderating mitochondrial translation, which were not restored with short‐term recovery, but in many cases were restored with long‐term recovery

**FIGURE 5:**
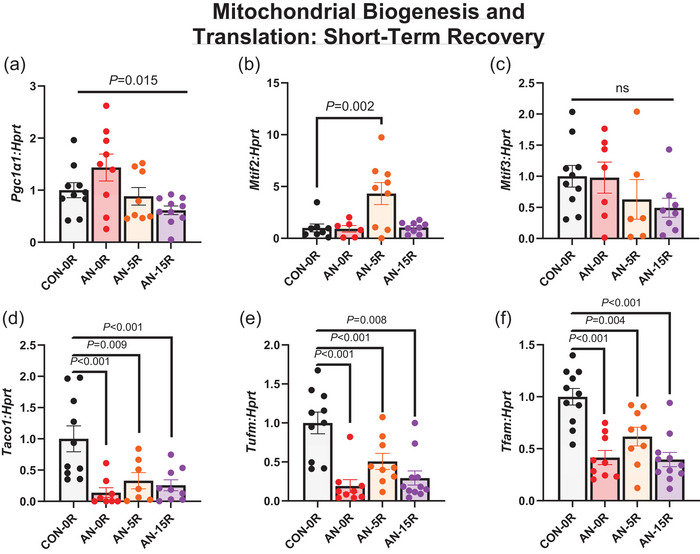
mRNA levels for genes related to mitochondrial biogenesis and translation from experiment 2 (short‐term recovery). mRNA levels of: (a) *Pgc1α1*, (b) *Mtif2*, (c) *Mtif3*, (d) *Taco1*, (e) *Tufm*, and (f) *Tfam*. Data are depicted as means ± SEM. Data were analysed by one‐way ANOVA with Dunnett's *post hoc* adjustment.

The global *F*‐test for the mitochondrial biogenesis marker, *Pgc1α1*, was significant (*P =* 0.015); however, no pairwise comparisons reached statistical significance (*P =* 0.188–0.936, Figure 5a). Regarding genes responsible for moderating translation of the mitochondrial‐specific genome, *Mtif2* was 3.3‐fold greater in AN‐5R compared to CON‐0R (*P =* 0.002), without any differences between CON‐0R and AN‐0R (*P =* 0.999,) or CON‐0R and AN‐15R (*P =* 0.999, Figure 5b). There were no differences between any groups in mRNA levels of *Mtif3* (*P =* 0.211–0.999, Figure 5c). *Taco1* mRNA levels were 85% lower in AN‐0R compared to CON‐0R (*P <* 0.001) and were also 67% and 74% lower than in AN‐5R and AN‐15R, respectively (*P =* 0.009 and *P <* 0.001, Figure 5d). *Tufm* mRNA levels had a similar pattern, with AN‐0R rats having 80% lower levels of *Tufm* mRNA compared to CON‐0R (*P <* 0.001), which remained 50% and 71% lower in AN‐5R and AN‐15R respectively (*P <* 0.001 and *P =* 0.008, Figure 5E). Finally, *Tfam* mRNA levels were also 58% lower in AN‐0R compared to CON‐0R and continued to be 38% and 60% lower in AN‐5R and AN‐15R, respectively (*P =* 0.004 and *P <* 0.001, Figure 5f).

**FIGURE 6:**
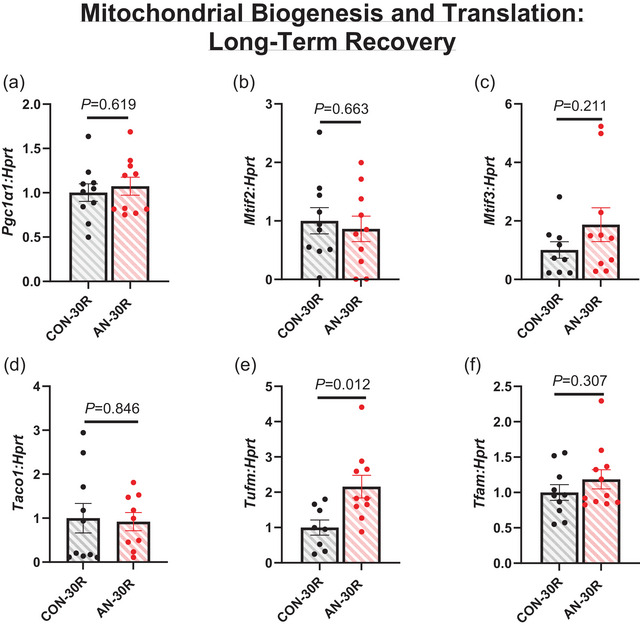
mRNA levels for genes related to mitochondrial biogenesis and translation from experiment 3 (long‐term recovery). mRNA levels of: (a) *Ppargc1a*, (b) *Mtif2*, (c) *Mtif3*, (d) *Taco1*, (e) *Tufm*, and (f) *Tfam*. Data are depicted as means ± SEM. Data were analysed by *t*‐test.

Among long‐term recovery rats, there were no differences between CON‐30R and AN‐30R in *Pgc1α1* (*P =* 0.619), *Mtif2* (*P =* 0.663), *mtiF3* (*P =* 0.211), *Taco1* (*P =* 0.846), or *Tfam* mRNA levels (*P =* 0.307, Figure 6a–d, f). However, regarding *Tufm*, AN‐30R rats had ∼1.1‐fold greater *Tufm* mRNA levels compared to CON‐30R (*P =* 0.012, Figure 6e).

### 3.5 Experiment 2: Simulated AN altered mRNA markers for mitochondrial dynamics, many of which were not restored with either short‐ or long‐term recovery

**FIGURE 7:**
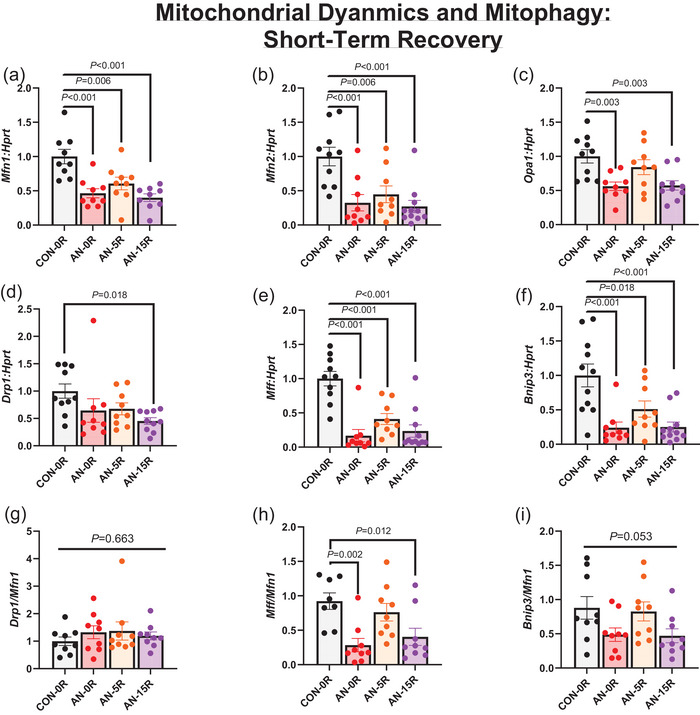
mRNA levels for genes related to mitochondrial dynamics and mitophagy from experiment 2 (short‐term recovery). mRNA levels of: (a) *Mfn1* (fusion), (b) *Mfn2* (fusion), (c) *Opa1* (fusion), (d) *Drp1* (fission), (e) *Mff* (fission), (f) *Bnip3* (mitophagy), and (g) *Drp1/Mfn1*, (h) *Mff/Mfn1*, (i) *Bnip3/Mfn1*. Data are depicted as means ± SEM. Data were analysed by one‐way ANOVA with Dunnett's *post hoc* adjustment.

Mitochondrial fusion markers, *Mfn1*, *Mfn2* and *Opa1*, were altered with AN and subsequent recovery. Specifically, AN‐0R rats had 50% lower *Mfn1* compared to CON‐0R (*P <* 0.001), which remained 40% and 60% lower in AN‐5R and AN‐15R rats (*P =* 0.006 and *P <* 0.001, Figure 7a). Additionally, AN‐0R rats had 65% lower *Mfn2* mRNA levels compared to CON‐0R (*P <* 0.001), and AN‐5R and AN‐15R also had 55% and 70% lower *Mfn2* mRNA levels compared to CON‐0R (*P =* 0.006 and *P <* 0.001, respectively, Figure 7b). *Opa1* mRNA levels were also 40% lower in AN‐0R compared to CON‐0R (*P =* 0.003) and, although not different in AN‐5R (*P =* 0.446), were 40% lower in AN‐15R compared to CON‐0R (*P =* 0.003, Figure 7c).

Mitochondrial fission and mitophagy markers were also altered by AN. While the global *P*‐value was significant for mitochondrial fission marker *Drp1* (*P =* 0.049), the only pairwise difference that reached significance was AN‐15R compared to CON‐0R (*P =* 0.018, Figure 7d). Mitochondrial fission marker *Mff* was 83% lower in AN‐0R compared to CON‐0R (*P <* 0.001), which continued to be 55% and 75% lower in AN‐5R and AN‐15R compared to CON‐0R, respectively (*P <* 0.001 and *P <* 0.001, Figure 7e). The mitophagy marker, *Bnip3*, was 75% lower in AN‐0R compared to CON‐0R (*P <* 0.001), and this difference was sustained in AN‐5R and AN‐15R rats, with ∼45% and ∼75% lower *Bnip3* mRNA levels compared to CON‐0R, respectively (*P =* 0.018 and *P <* 0.001, Figure 7f).

To estimate a global mitochondrial fusion/fission balance, we normalized mitochondrial fission and mitophagy markers to *Mfn1* mRNA levels, as Mfn1 is a key regulator of mitochondrial fusion. There were no differences in *Drp1/Mfn1* content across any AN or recovery groups compared to CON‐0R (*P =* 0.663, Figure 7g). However, there was 63% lower *Mff/Mfn1* ratio in AN‐0R compared to CON‐0R (*P =* 0.002, Figure 7h). This ratio was also 52% lower in AN‐15R compared to CON‐0R (*P =* 0.012) with no differences between AN‐5R and CON‐0R (*P =* 0.658, Figure 7h). Finally, the global *F*‐test for the ratio of *Bnip3/Mfn1* approached statistical significance (*P =* 0.053), but there were no significant differences in pairwise comparisons (*P =* 0.081–0.982, Figure 7i). Data showed similar patterns when normalized to *Mfn2* mRNA levels (data not shown).

### 3.6 Experiment 3: mRNA markers of mitochondrial dynamics and mitophagy were altered in AN rats after long‐term recovery

**FIGURE 8:**
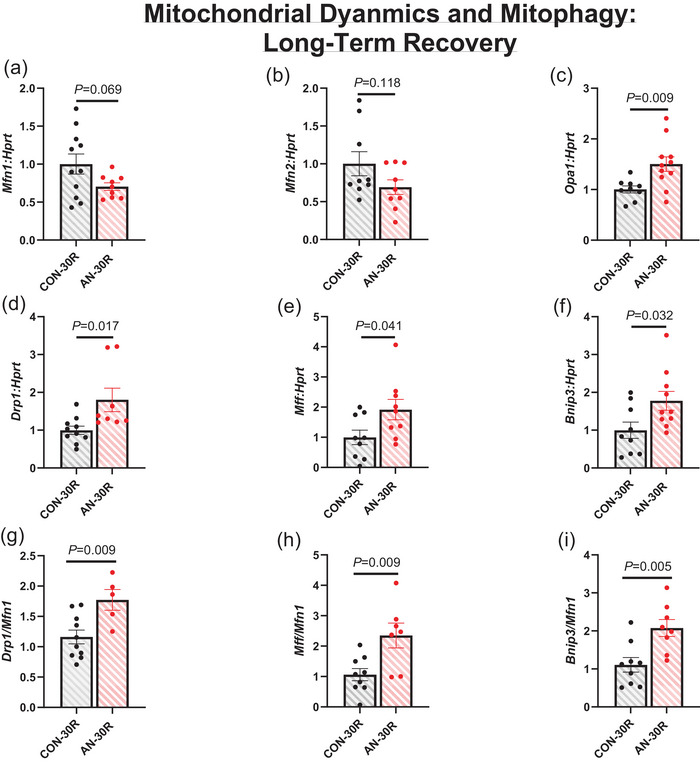
mRNA levels for genes related to mitochondrial dynamics and mitophagy from experiment 3 (long‐term recovery). mRNA levels of: (a) *Mfn1* (fusion), (b) *Mfn2* (fusion), (c) *Opa1* (fusion), (d) *Drp1* (fission), (e) *Mff* (fission), (f) *Bnip3* (mitophagy), (g) *Drp1/Mfn1*, (h) *Mff/Mfn1* and (i) *Bnip3/Mfn1*. Data are depicted as means ± SEM. Data were analysed by *t*‐test.

mRNA levels of mitochondrial fusion markers, *Mfn1* and *Mfn2*, were 30% lower in AN‐30R compared to CON‐30R, but these differences did not reach statistical significance (*P =* 0.069 and *P =* 0.118, Figure 8a, b). *Opa1* mRNA levels were 50% greater at AN‐30R, compared CON‐30R (*P =* 0.009, Figure 8c). mRNA levels of the mitochondrial fission marker, *Drp1*, were 80% greater in AN‐30R compared to CON‐30R (*P =* 0.017, Figure 8d), and *Mff* mRNA levels were 90% greater in AN‐30R compared to CON‐30R (*P =* 0.041, Figure 8e). mRNA levels of the mitophagy marker, *Bnip3*, were 75% greater in AN‐30R compared to CON‐30R (*P =* 0.032, Figure 8f). When evaluating the balance between mitochondrial fission markers relative to fusion markers, AN‐30R rats had a *Drp1*/*Mfn1* ratio 60% greater than did CON‐30R rats (*P =* 0.009, Figure 8g), as well as an 135% greater ratio of *Mff/Mfn1* mRNA levels (*P =* 0.009, Figure 8h). Lastly, AN‐30R rats had a 2‐fold greater ratio of *Bnip3/Mfn1* mRNA levels compared to CON‐30R rats (*P =* 0.005, Figure 8i). Data showed similar patterns when normalized to *Mfn2* mRNA levels (data not shown).

## 4 DISCUSSION

This study evaluated multiple components of skeletal muscle mitochondrial respiratory capacities and quality control mechanisms in a model of AN and after several durations of weight gain following food restriction. We found that simulated AN reduces muscle performance during a fatiguing protocol, decreases utilization of complex I of the mitochondrial electron transfer system, and alters genes related to mitochondrial quality control. Moreover, short‐term weight gain does not normalize many of these changes. Given sufficient recovery time (e.g., duration of recovery matching duration of AN), some facets of muscle function and mitochondrial quality control are restored; however, skeletal muscle mRNA profiles seem to shift to favour mitochondrial fission, suggesting that changes in muscle biology resulting from severe calorie restriction may be more persistent than previously thought.

We found that simulated AN was sufficient to result in reduced submaximal force production during a fatiguing protocol, corresponding to lower overall muscle performance. Although this specific functional measurement has not been reported in patients with AN, reduced endurance (as assessed by aerobic fitness) is often present in those with AN (Minano‐Garrido et al., 2025; Tokumura et al., 2003) and remains lower even after short‐term weight gain (Minano‐Garrido et al., 2025). This finding is aligned with our prior work in rodents finding lower muscle strength, which was not restored with weight gain (Rosa‐Caldwell et al., 2025). Notably, changes in submaximal muscle performance during a fatiguing protocol are resistant to weight gain, with AN‐5R and AN‐15R rats having lower fatigue AUC and lower peak force compared to healthy controls. Given sufficient recovery time, muscle function does appear to recover to levels of healthy controls (AN‐30R rats), but this process is prolonged and likely requires sustained weight restoration. AN often has high relapse rates (>25%) (Khalsa et al., 2017); thus, this finding of restoration of muscle function is promising, but needs to be interpreted with caution. We do not know how long these muscle function parameters would take to recover following multiple episodes of relapse/recovery. While submaximal muscle performance appears to recover in these rats, as noted in our previous investigation (Rosa‐Caldwell et al., 2025), multiple facets of maximal muscle strength (e.g., grip strength and maximal plantar flexion strength) are not fully restored in AN‐30R rats to levels in healthy controls (Rosa‐Caldwell et al., 2025). Taken together, the adverse musculoskeletal effects of AN persist longer than previously thought.

In experiment 1, we found the contribution of complex I‐supported oxidative phosphorylation to maximal non‐coupled electron transfer is lower in rats with simulated AN compared to healthy controls, but there was no difference in either phosphorylating or non‐phosphorylating respiration supported by complex I and II (relative to tissue wet weight). To our knowledge, mitochondrial function in the skeletal muscle following severe, sustained energy deprivation (simulated AN) has not been reported previously. Multiple prior studies of muscle atrophy have noted changes in mitochondrial respiration (Brown et al., 2017; Delfinis et al., 2022; Fukunaga et al., 2021; Rahman et al., 2025; Wang et al., 2018), and recent work suggests mitochondrial dysfunction as a key driving factor in AN (Xu et al., 2025). Moreover, in some models of muscle atrophy, such as cancer‐induced muscle loss (e.g., cachexia), changes in mitochondrial function are believed to be initiating factors in the development of muscle atrophy and strong moderators of overall disease progression (Brown et al., 2017; Delfinis et al., 2022). Complex I specifically is implicated in a number of diseases, with several neurological diseases characterized by mutations in mtDNA‐encoded subunits of complex I (Vartak et al., 2015). Importantly, of the five complexes of the mitochondrial electron transfer system, only complex II is entirely encoded by the nuclear genome, with the other four complexes containing subunits encoded by both the nuclear and mitochondrial genome (Fullerton et al., 2020). Our data show losses of gene expression related to mitochondrial transcription with simulated AN (discussed further below). Correspondingly, our respiration data suggest possible maintenance of and/or compensation by complex II due to deficits in complex I, which may be related to downregulated or dysfunctional mtDNA‐encoded subunits of complex I. We cannot speculate if these changes in complex utilization cause muscle loss during the development of AN or if they are a consequence of severe calorie deprivation, nor can we predict if these changes in mitochondria persist even after weight regain. As such, future investigation should evaluate the potential mechanistic role of mitochondria in the development of AN‐induced muscle atrophy and how weight restoration may affect changes in mitochondrial respiration following AN.

The decreased reliance on complex I may be an attempt to counteract elevated reactive oxygen species (ROS) production. Complexes I and III and their accompanied pathways (NADH/NAD^+^ for complex I, ubiquinone for complex III) are most associated with ROS production, especially at rest (Brand, 2016). A current hypothesis is that greater reliance on complex II over complex I may decrease damaging ROS production. Additionally, complex II (also known as succinate dehydrogenase) obtains electrons from FADH_2_ as opposed to NADH (complex I), so the greater reliance on complex II may indicate a switch in preference of substrate utilization to fat, which generates more FADH_2_ through β‐oxidation, than carbohydrates, which generate primarily NADH during glycolysis. That said, complex I tends to be more adaptable to exercise training, so these preliminary data generate exciting new frontiers of discovery within AN.

In both experiments 2 and 3, we found changes in expression of genes moderating mitochondrial translation, which were not resolved with short‐term weight recovery. For example, we noted reductions in mRNA levels of the genes *Taco1*, *Tufm* and *Tfam*. Mitochondria have their own unique encoded genome and the translation of such genes is key for maintenance of mitochondrial respiration and ATP production (D'Souza & Minczuk, 2018). To our knowledge, these musculoskeletal alterations during acute AN have not been reported previously. However, in other models of muscle atrophy, particularly in disuse atrophy, the expression of many of these genes is markedly decreased in the development and progression of disuse‐induced muscle loss (de Smalen et al., 2023; Rosa‐Caldwell et al., 2019, 2021). Additionally, other studies have noted that reduction in mitochondrial translation directly corresponds to decreased mitochondrial respiration (Čunátová et al., 2024; Lee et al., 2019). Therefore, the changes in these genes suggest that, during sustained low weight AN, there may be decreased translation of the mitochondria‐specific genome that is not restored with short‐term weight gain. We speculate that these alterations in mitochondrial gene expression may in part explain differences in mitochondrial respiration, in particular the noted changes in complex I (Čunátová et al., 2024) found in experiment 1; however, more research directly evaluating mitochondrial respiration with weight restoration is needed to substantiate these claims.

Finally, we also noted changes in content of genes involved in mitochondrial dynamics (fusion/fission) as well as in mitophagy. For example, during AN and short‐term recovery (experiment 2), mRNA levels of genes encoding proteins involved in mitochondrial fusion (*Mfn1*, *Mfn2*, *Opa1*), fission (*Drp*, *Mff*) and mitophagy (*Bnip3*) were all lower than in healthy controls. However, the overall balance of fusion versus fission gene expression was not markedly changed. In contrast, with long‐term recovery, there was a shift towards increased mitochondrial fission and mitophagy, whereby AN‐30R rats had greater *Drp1*, *Mff* and *Bnip3* mRNA levels. All of these processes are known to be important for maintenance of the mitochondrial network; however, if the balance of these processes is shifted, remodelling of the mitochondrial network results. For example, exercise or short‐term fasting are known to shift mitochondrial dynamics towards fusion, thus generating a more efficient mitochondrial network (Lee et al., 2014; Li et al., 2025; Ruegsegger et al., 2023). In contrast, during pathological conditions such as insulin resistance or muscular dystrophy, excessive mitochondrial fission is thought to contribute to a fragmented and less efficient mitochondrial network (Jheng et al., 2012; Rosen et al., 2025). Based on our current data, it seems plausible that during AN and short‐term weight gain, mitochondrial dynamics and mitophagy are reduced in a compensatory effort to maintain the mitochondrial pool; however, with long‐term recovery, the mitochondrial dynamics shift to favour greater mitochondrial fission. Whether this shift is physiological to restore mitochondrial health or pathological to result in long‐term muscle complications is not known. Given the residual effects on muscle strength that we have noted previously in these animals (Rosa‐Caldwell et al., 2025), it is possible the shift towards increased mitochondrial fission may contribute to the long‐term reduction in muscle strength. However, more data are necessary to evaluate this hypothesis.

There are a few limitations to this that should be acknowledged. First, this rodent model of AN does not capture the complex psychological sequela inherent to human AN and it is possible the voluntary nature of food restriction during AN (as opposed to forced food restriction in the present study) may be contributing in unknown ways to physiological outcomes. Additionally, due to the age of these rodents it is difficult to directly assess muscle atrophy and loss as opposed to attenuation of maturation and development. Given our data on muscle size, we expect our model of AN (and indeed most human manifestations of AN) has a combination of both; however, at this point we cannot determine precisely the relative contribution of each. It is also possible some of the more subtle differences we noted in groups during recovery may be due to differences in muscle water content, due to changes in glycogen and subsequent water storage. While we expect most of the differences in muscle size are due to loss of contractile units, water storage in the muscle warrants further investigation. Moreover, we did not directly assess mitochondrial density or volume. There may have been alterations in mitochondrial volume density that, ultimately, did not impact the overall capacity of the mitochondria within a given unit of muscle mass. Given that coupling efficiency (FCR_PCI+II_) appeared to be lower with AN (*P =* 0.088), it is possible that more mitochondria are needed to perform a comparable amount of work in AN so that if mitochondrial capacities were normalized to mitochondrial volume density, capacities may be lower per mitochondrial unit. Finally, we did not measure protein content for our mRNA target genes, so we do not know if the transcriptional changes we noted across experiments translated to differences at the protein level.

In conclusion, simulated AN resulted in profound changes to submaximal muscle performance during a fatiguing protocol, which coincides with reductions in mitochondrial reliance on complex I. Moreover, various genes responsible for mitochondrial quality control processes are also altered in AN. Many of these changes are not resolved with short‐term weight gain, including muscle fatigability, mitochondrial translation, and mitochondrial dynamics and mitophagy. However, after long‐term, sustained weight gain, matching the duration of AN, muscle endurance and many facets of mitochondrial quality control mechanisms seem to normalize. The notable exception was a shift towards greater mitochondrial fission, which may partially explain some long‐term changes in muscle strength noted in association with AN. Future studies should further evaluate these mitochondrial alterations and how they may be contributing to development of AN and/or long‐term AN‐induced musculoskeletal impairments.

## AUTHOR CONTRIBUTIONS

Conception or design of work (Megan E. Rosa‐Caldwell, Ursula B. Kaiser, Seward B. Rutkove, Kevin A. Murach, Lauren Breithaupt), acquisition, analysis or interpretation of data for the work (Megan E. Rosa‐Caldwell, Toby L. Chambers, Lauren Breithaupt, Ruqaiza Muhyudin, Patience Salvalina Okoto, Sadie R. Thompson, Emily E. Rothacker, Claire Greenhill, Katie A. Wood, Kevin A. Murach, Sarah H. White‐Springer, Ursula B. Kaiser, Seward B. Rutkove), drafting the work or revising it critically for important intellectual content (Megan E. Rosa‐Caldwell, Toby L. Chambers, Lauren Breithaupt, Ruqaiza Muhyudin, Patience Salvalina Okoto, Sadie R. Thompson, Emily E. Rothacker, Claire Greenhill, Katie A. Wood, Kevin A. Murach, Sarah H. White‐Springer, Ursula B. Kaiser, Seward B. Rutkove). All authors confirm they have approved the final version of the manuscript, agree to be accountable for all aspects of the work in ensuring that questions related to the accuracy or integrity of any part of the work are appropriately investigated and resolved, all persons designated as authors qualify for authorship, and all those who qualify for authorship are listed.

## CONFLICT OF INTEREST

The authors declare they have no conflicts of interest.

## Data Availability

Raw data and associated statistical code are available at our Open Science Framework page for this project at: https://doi.org/10.17605/OSF.IO/GJ5EF

## References

[eph70146-bib-0001] Arcelus, J. , Mitchell, A. J. , Wales, J. , & Nielsen, S. (2011). Mortality rates in patients with anorexia nervosa and other eating disorders. A meta‐analysis of 36 studies. Archives of General Psychiatry, 68(7), 724–731. 21727255 10.1001/archgenpsychiatry.2011.74

[eph70146-bib-0002] Artman, J. L. , Wesolowski, L. T. , Semanchik, P. L. , Isles, J. K. , Norton, S. A. , & White‐Springer, S. H. (2024). Local and systemic responses to repeated gluteal muscle microbiopsies in mature sedentary horses. Journal of Equine Veterinary Science, 136, 105070. 38642813 10.1016/j.jevs.2024.105070

[eph70146-bib-0003] Bhasin, H. , O'Brien, S. C. , Cordner, Z. A. , Aston, S. A. , Tamashiro, K. L. K. , & Moran, T. H. (2023). Activity‐based anorexia in adolescent female rats causes changes in brain mitochondrial dynamics. Physiology & Behavior, 261, 114072. 36599403 10.1016/j.physbeh.2022.114072

[eph70146-bib-0004] Brand, M. D. (2016). Mitochondrial generation of superoxide and hydrogen peroxide as the source of mitochondrial redox signaling. Free Radical Biology and Medicine, 100, 14–31. 27085844 10.1016/j.freeradbiomed.2016.04.001

[eph70146-bib-0005] Brown, J. L. , Rosa‐Caldwell, M. E. , Lee, D. E. , Blackwell, T. A. , Brown, L. A. , Perry, R. A. , Haynie, W. S. , Hardee, J. P. , Carson, J. A. , Wiggs, M. P. , Washington, T. A. , & Greene, N. P. (2017). Mitochondrial degeneration precedes the development of muscle atrophy in progression of cancer cachexia in tumour‐bearing mice. Journal of Cachexia Sarcopenia Muscle, 8(6), 926–938. 28845591 10.1002/jcsm.12232 PMC5700433

[eph70146-bib-0006] Čunátová, K. , Vrbacký, M. , & Puertas‐Frias, G. (2024). Mitochondrial translation is the primary determinant of secondary mitochondrial complex I deficiencies. Iscience, 27(8), 110560. 39184436 10.1016/j.isci.2024.110560 PMC11342289

[eph70146-bib-0007] Delfinis, L. J. , Bellissimo, C. A. , Gandhi, S. , Dibenedetto, S. N. , Garibotti, M. C. , Thuhan, A. K. , Tsitkanou, S. , Rosa‐Caldwell, M. E. , Rahman, F. A. , Cheng, A. J. , Wiggs, M. P. , Schlattner, U. , Quadrilatero, J. , Greene, N. P. , & Perry, C. G. R. (2022). Muscle weakness precedes atrophy during cancer cachexia and is linked to muscle‐specific mitochondrial stress. JCI Insight, 7(24), e155147. 36346680 10.1172/jci.insight.155147 PMC9869968

[eph70146-bib-0008] de Smalen, L. M. , Börsch, A. , & Leuchtmann, A. B. (2023). Impaired age‐associated mitochondrial translation is mitigated by exercise and PGC‐1α. Proceedings of the National Academy of Sciences of the United States of America, 120(36), e2302360120. 37639610 10.1073/pnas.2302360120 PMC10483666

[eph70146-bib-0009] D'Souza, A. R. , & Minczuk, M. (2018). Mitochondrial transcription and translation: Overview. Essays in Biochemistry, 62(3), 309–320. 30030363 10.1042/EBC20170102 PMC6056719

[eph70146-bib-0010] Eddy, K. T. , Tabri, N. , Thomas, J. J. , Murray, H. B. , Keshaviah, A. , Hastings, E. , Edkins, K. , Krishna, M. , Herzog, D. B. , Keel, P. K. , & Franko, D. L. (2017). Recovery from anorexia nervosa and bulimia nervosa at 22‐year follow‐up. The Journal of Clinical Psychiatry, 78(02), 184–189. 28002660 10.4088/JCP.15m10393 PMC7883487

[eph70146-bib-0011] Fukunaga, T. , Mori, S. , Omura, T. , Noda, Y. , Fujita, Y. , Ohsawa, I. , & Shigemoto, K. (2021). Muscle fiber type specific alterations of mitochondrial respiratory function and morphology in aged female mice. Biochemical and Biophysical Research Communications, 540, 116–122. 33472133 10.1016/j.bbrc.2020.11.071

[eph70146-bib-0012] Fullerton, M. , McFarland, R. , Taylor, R. W. , & Alston, C. L. (2020). The genetic basis of isolated mitochondrial complex II deficiency. Molecular Genetics and Metabolism, 131(1–2), 53–65. 33162331 10.1016/j.ymgme.2020.09.009 PMC7758838

[eph70146-bib-0013] Gaiaschi, L. , Priori, E. C. , Mensi, M. M. , Verri, M. , Buonocore, D. , Parisi, S. , Hernandez, L. N. Q. , Brambilla, I. , Ferrari, B. , De Luca, F. , Gola, F. , Rancati, G. , Capone, L. , Andriulo, A. , Visonà, S. D. , Marseglia, G. L. , Borgatti, R. , & Bottone, M. G. (2024). New perspectives on the role of biological factors in anorexia nervosa: Brain volume reduction or oxidative stress, which came first? Neurobiology of Disease, 199, 106580. 38942323 10.1016/j.nbd.2024.106580

[eph70146-bib-0014] Galmiche, M. , Déchelotte, P. , Lambert, G. , & Tavolacci, M. P (2019). Prevalence of eating disorders over the 2000–2018 period: A systematic literature review. The American Journal of Clinical Nutrition, 109(5), 1402–1413. 31051507 10.1093/ajcn/nqy342

[eph70146-bib-0015] Greene, N. P. , Lee, D. E. , Brown, J. L. , Rosa, M. E. , Brown, L. A. , Perry, R. A. , Henry, J. N. , & Washington, T. A. (2015). Mitochondrial quality control, driven by PGC‐1α, is dysregulated by Western Diet‐induced obesity and partially restored by moderate physical activity in mice. Physiological Reports, 3(7), e12470. 26177961 10.14814/phy2.12470 PMC4552545

[eph70146-bib-0016] Hudson, J. I. , Hiripi, E. , Pope, H. G., Jr , & Kessler, R. C. (2007). The prevalence and correlates of eating disorders in the National Comorbidity Survey Replication. Biological Psychiatry, 61(3), 348–358. 16815322 10.1016/j.biopsych.2006.03.040 PMC1892232

[eph70146-bib-0017] Jheng, H. F. , Tsai, P. J. , Guo, S. M. , Kuo, L. H. , Chang, C. S. , Su, I. J. , Chang, C. R. , & Tsai, Y. S. (2012). Mitochondrial fission contributes to mitochondrial dysfunction and insulin resistance in skeletal muscle. Molecular and Cellular Biology, 32(2), 309–319. 22083962 10.1128/MCB.05603-11 PMC3255771

[eph70146-bib-0018] Khalsa, S. S. , Portnoff, L. C. , McCurdy‐McKinnon, D. , & Feusner, J. D. (2017). What happens after treatment? A systematic review of relapse, remission, and recovery in anorexia nervosa. Journal of Eating Disorders, 5(1), 20. 28630708 10.1186/s40337-017-0145-3 PMC5470198

[eph70146-bib-0019] Lark, D. S. , Torres, M. J. , Lin, C. T. , Ryan, T. E. , Anderson, E. J. , & Neufer, P. D. (2016). Direct real‐time quantification of mitochondrial oxidative phosphorylation efficiency in permeabilized skeletal muscle myofibers. American Journal of Physiology Cell Physiology, 311(2), C239–C245. 27335172 10.1152/ajpcell.00124.2016 PMC5129772

[eph70146-bib-0020] Latham, C. M. , Fenger, C. K. , & White, S. H. (2019). Rapid communication: Differential skeletal muscle mitochondrial characteristics of weanling racing‐bred horses. Journal of Animal Science, 97(8), 3193–3198. 31211376 10.1093/jas/skz203 PMC6667244

[eph70146-bib-0021] Lee, D. E. , Perry, R. A. , Brown, J. L. , Rosa‐Caldwell, M. E. , Brown, L. A. , Haynie, W. S. , Rajaram, N. , Washington, T. A. , & Greene, N. P. (2019). Mitochondrial mRNA translation initiation contributes to oxidative metabolism in the myocardia of aged, obese mice. Experimental Gerontology, 121, 62–70. 30928679 10.1016/j.exger.2019.03.009

[eph70146-bib-0022] Lee, J. Y. , Kapur, M. , & Li, M. (2014). MFN1 deacetylation activates adaptive mitochondrial fusion and protects metabolically challenged mitochondria. Journal of Cell Science, 127, (Pt 22), 4954–4963. 25271058 10.1242/jcs.157321 PMC4231308

[eph70146-bib-0023] Li, Y. , Zhao, W. , & Yang, Q. (2025). Effects of high‐intensity interval training and moderate‐intensity continuous training on mitochondrial dynamics in human skeletal muscle. Frontiers in Physiology, 16, 1554222. 40313872 10.3389/fphys.2025.1554222 PMC12043657

[eph70146-bib-0024] Lindfors, C. , Nilsson, I. A. , & Garcia‐Roves, P. M. (2011). Hypothalamic mitochondrial dysfunction associated with anorexia in the anx/anx mouse. Proceedings of the National Academy of Sciences of the United States of America, 108(44), 18108–18113. 22025706 10.1073/pnas.1114863108 PMC3207677

[eph70146-bib-0025] Lock, J. , & Le Grange, D. (2019). Family‐based treatment: Where are we and where should we be going to improve recovery in child and adolescent eating disorders. The International Journal of Eating Disorders, 52(4), 481–487. 30520532 10.1002/eat.22980

[eph70146-bib-0026] Micali, N. , Martini, M. G. , Thomas, J. J. , Eddy, K. T. , Kothari, R. , Russell, E. , Bulik, C. M. , & Treasure, J. (2017). Lifetime and 12‐month prevalence of eating disorders amongst women in mid‐life: A population‐based study of diagnoses and risk factors. BMC Medicine [Electronic Resource], 15(1), 12. 28095833 10.1186/s12916-016-0766-4 PMC5240354

[eph70146-bib-0027] Minano‐Garrido, E. , Catalán‐Matamoros, D. , Acosta, G. P. , & Gómez‐Conesa, A. (2025). The effect of anorexia nervosa inpatient physiotherapy adapted program (ANIPAP): A clinical trial. Journal of Bodywork and Movement Therapies, 42, 337–343. 40325688 10.1016/j.jbmt.2024.12.043

[eph70146-bib-0028] Minnes, G. L. , Wiener, A. J. , & Pisahl, A. S. (2024). Effects of maternal separation on punishment‐driven risky decision making in adolescence and adulthood. Neurobiology of Learning and Memory, 217, 108016. 39709000 10.1016/j.nlm.2024.108016 PMC11769738

[eph70146-bib-0029] Mueller, S. M. , Immoos, M. , Anliker, E. , Drobnjak, S. , Boutellier, U. , & Toigo, M. (2015). Reduced bone strength and muscle force in women 27 years after anorexia nervosa. Journal of Clinical Endocrinology and Metabolism, 100(8), 2927–2933. 26086327 10.1210/jc.2015-1011

[eph70146-bib-0030] Omodei, D. , Pucino, V. , Labruna, G. , Procaccini, C. , Galgani, M. , Perna, F. , Pirozzi, D. , De Caprio, C. , Marone, G. , Fontana, L. , Contaldo, F. , Pasanisi, F. , Matarese, G. , & Sacchetti, L. (2015). Immune‐metabolic profiling of anorexic patients reveals an anti‐oxidant and anti‐inflammatory phenotype. Metabolism: Clinical and Experimental, 64(3), 396–405. 25500208 10.1016/j.metabol.2014.10.025

[eph70146-bib-0031] Rahman, F. A. , Graham, M. Q. , Adam, A. M. , Juracic, E. S. , Tupling, A. R. , & Quadrilatero, J. (2025). Mitophagy is required to protect against excessive skeletal muscle atrophy following hindlimb immobilization. Journal of Biomedical Science, 32(1), 29. 39979946 10.1186/s12929-025-01118-w PMC11844018

[eph70146-bib-0032] Rosa‐Caldwell, M. E. , Breithaupt, L. , Kaiser, U. B. , Garland, E. , Pinkham, S. , Hancock, M. , Jalkut, S. L. , & Rutkove, S. B. (2024). A refined rodent model of anorexia nervosa: Simulating state‐specific effects of caloric restriction and weight restoration. Physiological Reports, 12(12), e16092.

[eph70146-bib-0033] Rosa‐Caldwell, M. E. , Breithaupt, L. , Kaiser, U. B. , Muhyudin, R. , & Rutkove, S. B. (2025). Changes. in muscle strength and moderators of protein turnover in a rodent model of anorexia nervosa and recovery. Journal of Nutritional Physiology, 4, 100010.

[eph70146-bib-0034] Rosa‐Caldwell, M. E. , Brown, J. L. , Lee, D. E. , Blackwell, T. A. , Turner, K. W. , Brown, L. A. , Perry, R. A. , Haynie, W. S. , Washington, T. A. , & Greene, N. P. (2017). Autophagy activation, not peroxisome proliferator‐activated receptor gamma coactivator 1alpha, may mediate exercise‐induced improvements in glucose handling during diet‐induced obesity. Experimental Physiology, 102(9), 1194–1207. 28639297 10.1113/EP086406

[eph70146-bib-0035] Rosa‐Caldwell, M. E. , Brown, J. L. , & Perry, R. A., Jr. (2019). Regulation of mitochondrial quality following repeated bouts of hindlimb unloading. Applied Physiology, Nutrition and Metabolism, 45, 264–274. 10.1139/apnm-2019-0218 PMC7370318 31340136

[eph70146-bib-0036] Rosa‐Caldwell, M. E. , Eddy, K. T. , Rutkove, S. B. , & Breithaupt, L. (2022). Anorexia nervosa and muscle health: A systematic review of our current understanding and future recommendations for study. The International Journal of Eating Disorders, 56(3), 483–500. 36529682 10.1002/eat.23878

[eph70146-bib-0037] Rosa‐Caldwell, M. E. , & Greene, N. P. (2019). Muscle metabolism and atrophy: Let's talk about sex. Biology of Sex Differences, 10(1), 43. 31462271 10.1186/s13293-019-0257-3 PMC6714453

[eph70146-bib-0038] Rosa‐Caldwell, M. E. , Lim, S. , Haynie, W. S. , Brown, J. L. , Lee, D. E. , Dunlap, K. R. , Jansen, L. T. , Washington, T. A. , Wiggs, M. P. , & Greene, N. P. (2021). Mitochondrial aberrations during the progression of disuse atrophy differentially affect male and female mice. Journal of Cachexia, Sarcopenia and Muscle, 12(6), 2056–2068. 34585846 10.1002/jcsm.12809 PMC8718086

[eph70146-bib-0039] Rosa‐Caldwell, M. E. , Mortreux, M. , Wadhwa, A. , Kaiser, U. B. , Sung, D. M. , Bouxsein, M. L. , & Rutkove, S. B (2023a). Influence of gonadectomy on muscle health in micro‐ and partial‐gravity environments in rats. Journal of Applied Physiology, 134(6), 1438–1449. 37102698 10.1152/japplphysiol.00023.2023 PMC10228673

[eph70146-bib-0040] Rosa‐Caldwell, M. E. , Mortreux, M. , Wadhwa, A. , Kaiser, U. B. , Sung, D. M. , Bouxsein, M. L. , & Rutkove, S. B. (2023b). Sex differences in muscle health in simulated micro‐ and partial‐gravity environments in rats. Sports Medicine and Health Science, 5(4), 319–328. 38314043 10.1016/j.smhs.2023.09.002 PMC10831389

[eph70146-bib-0041] Rosen, H. G. , Berger, N. J. , Hodge, S. N. , Fujishiro, A. , Lourie, J. , Kapadia, V. , Duzz, T. , Linden, M. A. , Jee, E. , Kim, J. , Kim, Y. , & Zou, K. (2025). Inhibition of mitochondrial fission protein Drp1 ameliorates skeletal myopathy in the D2‐mdx model of duchenne muscular dystrophy. American Journal of Physiology Cell Physiology, 329(1), C307–C324. 40522885 10.1152/ajpcell.01009.2024 PMC12291061

[eph70146-bib-0042] Ruegsegger, G. N. , Pataky, M. W. , Simha, S. , Robinson, M. M. , Klaus, K. A. , & Nair, K. S (2023). High‐intensity aerobic, but not resistance or combined, exercise training improves both cardiometabolic health and skeletal muscle mitochondrial dynamics. Journal of Applied Physiology, 135(4), 763–774. 37616334 10.1152/japplphysiol.00405.2023 PMC10642518

[eph70146-bib-0043] Smink, F. R. , van Hoeken, D. , & Hoek, H. W. (2013). Epidemiology, course, and outcome of eating disorders. Current Opinion in Psychiatry, 26(6), 543–548. 24060914 10.1097/YCO.0b013e328365a24f

[eph70146-bib-0044] Stice, E. , Marti, C. N. , & Rohde, P. (2013). Prevalence, incidence, impairment, and course of the proposed DSM‐5 eating disorder diagnoses in an 8‐year prospective community study of young women. Journal of Abnormal Psychology, 122(2), 445–457. 23148784 10.1037/a0030679 PMC3980846

[eph70146-bib-0045] Tokumura, M. , Yoshiba, S. , Tanaka, T. , Nanri, S. , & Watanabe, H. (2003). Prescribed exercise training improves exercise capacity of convalescent children and adolescents with anorexia nervosa. European Journal of Pediatrics, 162(6), 430–431. 12707771 10.1007/s00431-003-1203-1

[eph70146-bib-0046] Vartak, R. , Deng, J. , Fang, H. , & Bai, Y. (2015). Redefining the roles of mitochondrial DNA‐encoded subunits in respiratory Complex I assembly. Biochimica et Biophysica Acta, 1852(7), 1531–1539. 25887158 10.1016/j.bbadis.2015.04.008 PMC4433823

[eph70146-bib-0047] Victor, V. M. , Rovira‐Llopis, S. , Saiz‐Alarcon, V. , Sangüesa, M. C. , Rojo‐Bofill, L. , Bañuls, C. , Falcón, R. , Castelló, R. , Rojo, L. , Rocha, M. , & Hernández‐Mijares, A. (2014). Altered mitochondrial function and oxidative stress in leukocytes of anorexia nervosa patients. PLoS ONE, 9(9), e106463. 25254642 10.1371/journal.pone.0106463 PMC4177818

[eph70146-bib-0048] Wang, D. , Wei, L. , Yang, Y. , & Liu, H. (2018). Dietary supplementation with ketoacids protects against CKD‐induced oxidative damage and mitochondrial dysfunction in skeletal muscle of 5/6 nephrectomised rats. Skeletal Muscle, 8(1), 18. 29855350 10.1186/s13395-018-0164-z PMC5984473

[eph70146-bib-0049] Wang, H. , Xu, X. , Yang, Z. , & Zhang, T. (2025). Alterations of synaptic plasticity and brain oscillation are associated with autophagy induced synaptic pruning during adolescence. Cognitive Neurodynamics, 19(1), 2. 39749102 10.1007/s11571-024-10185-y PMC11688264

[eph70146-bib-0050] Wesolowski, L. T. , Simons, J. L. , Semanchik, P. L. , Othman, M. A. , Kim, J.‐H. , Lawler, J. M. , Kamal, K. Y. , & White‐Springer, S. H. (2023). The impact of SRT2104 on skeletal muscle mitochondrial function, redox biology, and loss of muscle mass in hindlimb unloaded rats. International Journal of Molecular Sciences, 24(13), 11135. 37446313 10.3390/ijms241311135 PMC10342025

[eph70146-bib-0051] Wu, H. , Feng, L. , Wu, H. , Wang, L. , Xu, H. , & Fu, F. (2024). Synergistic effects of PS‐NPs and Cd on ovarian toxicity in adolescent rats: Ferroptosis by induction of mitochondrial redox imbalance via the SIRT3‐SOD2/Gpx4 pathway. Ecotoxicology and Environmental Safety, 290, 117622. 39732061 10.1016/j.ecoenv.2024.117622

[eph70146-bib-0052] Xu, J. , Zhang, R. , Millischer, V. , Stiernborg, M. , Tume, C. E. , Mehdinia, S. , Barker, P. , Yilmaz, Z. , Gonçalves, V. F. , Lavebratt, C. , Landén, M. , O'Rahilly, S. , Bulik, C. M. , & Nilsson, I. A. K. (2025). Elevated plasma GDF15 combined with FGF21 suggests mitochondrial dysfunction in a subgroup of anorexia nervosa patients. Translational Psychiatry, 15(1), 215. 40562759 10.1038/s41398-025-03425-0 PMC12198413

[eph70146-bib-0053] Yan, Z. , Lira, V. A. , & Greene, N. P. (2012). Exercise training‐induced regulation of mitochondrial quality. Exercise and Sport Sciences Reviews, 40(3), 159–164. 22732425 10.1097/JES.0b013e3182575599 PMC3384482

